# The prognostic impact of right ventricular-pulmonary arterial coupling in heart failure: a systematic review and meta-analysis

**DOI:** 10.1007/s10741-023-10341-2

**Published:** 2023-08-28

**Authors:** Vasileios Anastasiou, Andreas S. Papazoglou, Dimitrios V. Moysidis, Stylianos Daios, Konstantinos Barmpagiannos, Thomas Gossios, Georgios K. Efthimiadis, Theodoros Karamitsos, Antonios Ziakas, Vasileios Kamperidis

**Affiliations:** First Department of Cardiology, Medical School, Aristotle University of Thessaloniki, AHEPA Hospital, St. Kiriakidi 1, Thessaloniki, GR 54636 Greece

**Keywords:** Right ventricular-pulmonary artery coupling, TAPSE/PASP, Heart failure, Risk stratification

## Abstract

The echocardiographic tricuspid annular plane systolic excursion/pulmonary artery systolic pressure (TAPSE/PASP) ratio is a non-invasive surrogate of right ventricular-pulmonary arterial (RV-PA) coupling which corresponds well with the respective invasively derived index. Recently, a wealth of observational data has arisen, outlining its prognostic value in heart failure (HF) patients. To systematically appraise and quantitatively synthesize the evidence of the prognostic value of TAPSE/PASP ratio in left-sided HF regardless of etiology or left ventricular ejection fraction. A systematic literature review was conducted in electronic databases to identify studies reporting the association of TAPSE/PASP ratio with outcomes in patients with HF and, when appropriate, a random-effects meta-analysis was conducted to quantify the unadjusted and adjusted hazard ratios [(a)HRs] for all-cause death and the composite outcome of all-cause death or HF hospitalization. Eighteen studies were deemed eligible encompassing 8,699 HF patients. The applied cut-off value for RV-PA uncoupling varied substantially from 0.27 to 0.58 mm/mmHg, and in most studies values lower than the applied cutoff conveyed dismal prognosis. Eleven studies reported appropriate data for meta-analysis. TAPSE/PASP reduction by 1 mm/mmHg was independently associated with all-cause death (pooled aHR=1.32 [1.06-1.65]; p=0.01; I^2^=56%) and the composite outcome (pooled aHR=3.48 [1.67-7.25]; p<0.001; I^2^=0%). When a TAPSE/PASP cutoff value of 0.36 mm/mmHg was applied it yielded independent association with all-cause death (pooled aHR=2.84 [2.22-3.64]; p<0.001; I^2^=82%). RV-PA coupling assessed by echocardiographic TAPSE/PASP ratio appears to be an independent outcome predictor for HF patients.

**Supplementary Information:**

The online version contains supplementary material available at 10.1007/s10741-023-10341-2.

## Introduction

Despite the ongoing advances in disease-modifying therapies for patients with heart failure (HF), they continue to suffer a substantial risk for recurrent HF hospitalizations and cardiovascular death [1]. Beyond medical management, promising interventional therapies have entered the clinical arena in an effort to modify the natural history of HF. However, the risk stratification of HF patients and the selection of appropriate interventional treatments in a well-timed manner remain challenging, raising the quest for widely available risk stratification tools. Right ventricular (RV) dysfunction has been recognized as an indicator of poor prognostic course, providing information over and above left ventricular (LV) dysfunction [2]. Additionally, the prognostic role of pulmonary hypertension, a common hemodynamic complication of long-standing elevation of LV filling pressure in HF, has been reported [3, 4].

RV-pulmonary arterial (PA) coupling has emerged as a novel, comprehensive index that allows evaluating RV function in relation to the underlying RV afterload [5]. This index can be readily assessed non-invasively by the ratio of two standard echocardiographic measurements: tricuspid annular plane systolic excursion (TAPSE) over pulmonary artery systolic pressure (PASP). Recently, a large body of evidence has arisen outlining the prognostic role of echocardiographically assessed RV-PA coupling in chronic [6] and acute [7] HF patients, as well as recipients of interventional HF treatments including cardiac resynchronization therapy (CRT) [8] and transcatheter repair of functional mitral regurgitation (MR) or tricuspid regurgitation [9, 10].

This systematic review and meta-analysis sought to aggregate, systematically appraise and quantitatively synthesize the existing literature in regard to the long-term prognostic value of RV-PA coupling, assessed non-invasively by the echocardiographic TAPSE/PASP ratio, in patients with left-sided HF regardless of etiology or left ventricular ejection fraction.

## Methods

### Search strategy

The study was prospectively registered on the PROSPERO registry (PROSPERO 2023 CRD42023417767, https://www.crd.york.ac.uk/prospero/display_record.php?ID=CRD42023417767). A systematic electronic literature search was conducted up to April 10, 2023 using the MEDLINE, Scopus and Cochrane databases according to the Preferred Reporting Items for Systematic Reviews and Meta-Analyses guidelines (Supplemental Table 1) [11]. The Medical Subject Headings and keywords used as search terms were (‘’right ventricular-pulmonary arterial coupling’’ OR ‘’right ventricular-vascular coupling’’ OR ‘’tricuspid annular plane systolic excursion to pulmonary artery systolic pressure ratio’’ OR‘’TAPSE to PASP ratio’’) AND (‘’heart failure’’ OR ‘’functional mitral regurgitation’’ OR ‘’functional tricuspid regurgitation’’ OR ‘’prognosis’’ OR ‘’outcomes’’). Relevant reviews and the reference lists of the included studies were hand-searched to identify any non-detected relevant studies.

**Table 1:** Baseline characteristics of included studies (18 studies)

**Author**	**Year**	**Design**	**Included in meta-analysis**	**HF population**	**No. of patients**	**LVEF cut-off for inclusion, %**	**LVEF, % (mean)**	**Age, years, (mean)**	**Male, %**	**TAPSE/PASP, mm/mmHg (mean)**	**Vendor**	**Reproducibility for TAPSE/PASP**
Guazzi et al. [5]	2013	Prospective	Yes	HFrEF and HFpEF	293	-	36.0 ± 11.1	62.9 ± 8.9	79.2	0.50 ± 0.15	Phillips	Interobserver: coefficient of variation 3.5% (TAPSE), 4.7% (PASP); intraobserver: coefficient of variation 3% (TAPSE), 4% (PASP)
Guazzi et al. [28]	2015	Prospective	No	HFrEF and HFpEF	458	-	33.6 ± 10.6	62.5 ± 9.5	84.3	0.51 ± 0.17	Phillips	Interobserver: coefficient of variation 3.5% (TAPSE), 4.7% and 4.3 (PASP)
Iacoviello et al. [6]	2017	Retrospective	Yes	Stable HF, LVEF <45%	315	<45	NA	64.0 ±14.0	77.0	0.61 ± 0.23	GE	NA
Guazzi et al. [23]	2017	Prospective	No	HFpEF	387	≥50	59.5 ± 6.8	65.1 ± 11.5	40.4	NA	Phillips, GE	NA
Ghio et al. [14]	2017	Retrospective	Yes	Ischaemic, hypertensive, idiopathic HFrEF and HFpEF	1663	-	NA	65.0 ± 13.0	75.0	NA	NA	NA
Bosch et al. [20]	2017	Prospective	Yes	HFrEF and HFpEF	438	All	45.0 ± 8.0	66.5 ± 11	50.7	0.55 ± 0.27	NA	NA
Gorter et al. [18]	2018	Prospective	Yes	HF with LVEF ≥45% and suspected pulmonary hypertension	102	≥45	57.0 ± 5.0	73.4 ± 8.5	30.9	0.44 ± 0.20	GE	NA
Santas et al. [24]	2019	Prospective	No	Acute HFpEF	760	≥50	NA	75.6 ± 9.7	31.7	0.43 ± 0.17	Phillips	NA
Falletta et al. [15]	2019	Prospective	Yes	Clinically stable HFrEF	431	≤40	27.3 ± 5.7	59.0 ± 12.0	83.0	NA	GE	NA
Santas et al. [16]	2020	Prospective	Yes	Acute HFpEF	884	≥50	61.7 ±7.5	76.1 ± 9.7	35.6	0.44 ± 0.17	Phillips	NA
Braganca et al. [19]	2020	Retrospective	Yes	HFrEF undergoing CRT	70	<35	26.4 ± 7.1	69.0 ± 9.0	68.6	0.48 ± 0.24	Phillips	NA
Rosa et al. [26]	2020	Retrospective	No	HFrEF and HFmrEF	400	≤50	32.9 ± 8.5	77.5 ± 4.8	73.3	0.44 ± 0.33	Mountain View, Phillips	NA
Palazzuoli et al. [7]	2020	Prospective	No	Acute HFrEF and HFpEF	381	-	45.0 ± 11.0	81.5 ± 9.0	42.0	0.43 ± 0.31	NA	95% reproducibility
Schmeisser et al. [27]	2021	Prospective	No	HFrEF with indication for CRT	330	≤35	31.6 ± 3.4	66.4 ± 4.6	NA	0.39 ± 0.16	Phillips	NA
Karam et al. [9]	2021	Retrospective	No	Secondary MR undergoing TMVR	817	-	35.8 ± 13.1	73.8 ±10.1	66.3	0.46 ± 0.20	NA	Interobserver: interclass correlation coefficient >0.85
Deaconu et al. [22]	2021	Prospective	Yes	HFrEF undergoing CRT	54	<35	28.4 ± 1.3	64.0 ± 13.8	58.0	0.70 ± 0.20	NA	NA
Ishiwata et al. [21]	2021	Retrospective	Yes	Dilated cardiomyopathy, HFrEF	109	<40	22.0 ± 7.4	44.1 ± 14.0	69.8	0.47 ± 0.22	NA	NA
Stassen et al. [8]	2022	Retrospective	Yes	HFrEF undergoing CRT	807	≤35	27.8 ± 8.3	65.5 ± 10.5	76.0	0.46 ± 0.34	GE	NA

*CRT* cardiac resynchronization therapy, *GE* general electrics, *HF* heart failure, *HFmrEF* heart failure mildly reduced ejection fraction, *HFpEF* heart failure preserved ejection fraction, *HFrEF* heart failure reduced ejection fraction, *LVEF* left ventricular ejection fraction, *MR* mitral regurgitation, *NA* not available, *TAPSE/PASP* tricuspid annular plane systolic excursion/pulmonary artery systolic pressure, *TMVR* transcatheter mitral valve repair, *TR* tricuspid regurgitation, *TTVR* transcatheter tricuspid valve repair

### Study selection – eligibility criteria

The retrieved studies were independently assessed for eligibility by two investigators (V.A., K.B.), according to prespecified eligibility criteria. The eligibility criteria included all studies investigating the long-term prognostic significance of TAPSE/PASP ratio in left-sided HF patients with or without concomitant functional valvular heart disease, irrespective of LV ejection fraction or symptomatic status. Studies including organic or mixed valvulopathies were excluded. Also, studies including patients with functional tricuspid regurgitation of non left-sided etiology were deemed ineligible. TAPSE/PASP ratio was assessed both as categorical and continuous variable. Studies not reporting hazard ratios (HRs) or studies with suspected high risk of bias were not deemed eligible for the meta-analysis. Case reports, review papers, editorials, and letters were excluded.

### Data extraction

Data were independently extracted and reviewed by two investigators (V.A., K.B.). Any discrepancies were resolved by consensus with a third reviewer (S.D.). Pre-specified forms were used to extract epidemiological and clinical characteristics of the eligible studies. More specifically, the following data were extracted: study design, study population, demographic and echocardiographic data, follow-up period, outcomes of interest, association with outcomes and adjustment for confounding factors, whenever available.

### Risk of bias assessment

The methodological quality of the individual studies was assessed independently by two investigators (D.V.M. and S.D.) using the Quality In Prognosis Studies tool [12]. For each eligible study, the risk of bias was assessed as “low”, “moderate” or “high” in the following domains: study participation, study attrition, prognostic factor measurement, outcome measurement, study confounding, statistical analysis and reporting. Publication bias was not evaluated through the funnel plot method due to the limited number of eligible studies [13].

### Outcomes of interest

The primary study outcome was all-cause death, defined as death from any cause. The secondary study outcome was the composite of all-cause death or HF hospitalization.

### Data synthesis and statistical analysis

All unadjusted and adjusted HRs and the corresponding 95% confidence intervals of all-cause death and of the composite outcome were extracted for the TAPSE/PASP ratio, as a continuous variable, to reflect the risk difference per 1mm/mmHg of reduction. Moreover, the unadjusted and adjusted HRs and the corresponding 95% confidence intervals of all-cause death were extracted for the TAPSE/PASP ratio <0.36 mm/mmHg, as a binary variable. The value of 0.36 mm/mmHg was selected, as this cutoff was most commonly used in the eligible studies included in the meta-analysis [5, 14–16].

Pooled (a)HRs and 95% confidence intervals were computed using random-effect models (DerSimonian and Laird method) for both the primary and secondary outcome of interest, adjusted or unadjusted for clinical differences between the populations. A random effects model was selected *a priori* given the expected heterogeneity in study design across the eligible studies. Separate analyses using only unadjusted or adjusted data were conducted. Forest plots were constructed to show the overall effect of each parameter. The observed heterogeneity in each analysis was described using the I [2] statistic, which was quantified as low (<25%), moderate (25% to 75%), or high (>75%) [17].

All statistical analyses were performed using Review Manager (RevMan), Version 5.4, The Cochrane Collaboration, 2020. A two-tailed p-value of less than 0.05 was deemed as the statistical significance threshold for our study.

## Results

### Search outcomes

**Fig. 1:**
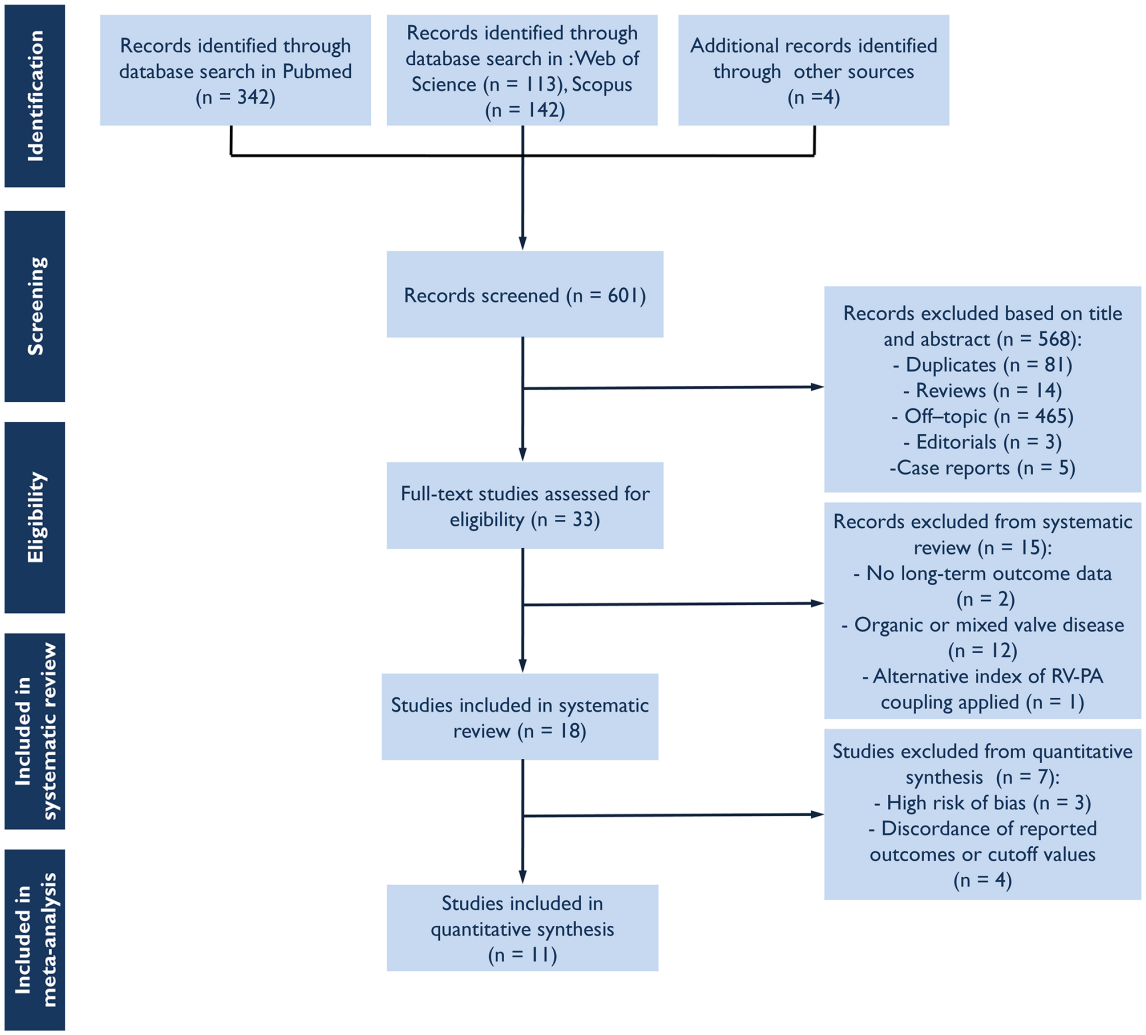
Study flow chart for study selection

The process of study selection is summarized in Fig. 1. From the initial 601 studies identified based on the search strategy, 18 relevant eligible full-text article were included in this systematic review. Of these, 11 were eligible for inclusion in the meta-analysis [5, 6, 8, 14–16, 18–22]. Four out of 7 studies were not included in the quantitative synthesis as they reported discordant outcomes and cutoff values [7, 23–25], whereas 3 out of 7 studies were deemed of prohibitive risk of bias (Supplemental Table S2) [26–28]. Overall, the risk of bias was considered to be low or moderate for the 11 studies included in meta-analysis (Supplemental Table S3).

### Qualitative analysis

#### Study characteristics

The baseline characteristics of the 18 studies included in the systematic review are summarized in Table 1. Overall, the studies included diverse HF cohorts based on the LV ejection fraction; 6 studies included HF patients with reduced ejection fraction [8, 15, 19, 21, 22, 27], 3 studies constituted of HF patients with preserved ejection fraction [3, 16, 24], and 9 studies encompassed HF subjects with mixed ejection fraction [5–7, 9, 14, 18, 20, 26, 28]. One out of the 9 studies with mixed ejection fraction investigated subjects with HF and functional mitral regurgitation (MR) receiving transcatheter repair [9]. Mean age of the study populations varied significantly from 44.1±14.0 to 81.5±9.0 years, while the number of enrolled patients ranged from 54 to 1663. The mean value of TAPSE/PASP ratio among studies varied from 0.34±0.50 to 0.70±0.20 mm/mmHg (Table 1).

### Prognostic value of TAPSE/PASP

Out of the 18 studies addressing the prognostic role of TAPSE/PASP in HF, 11 studies examined all-cause or cardiovascular mortality as the primary outcome [5, 6, 8, 9, 14–16, 18, 19, 26, 27], 1 study examined HF readmissions [24], and the rest included combined primary outcomes (Table 2) [7, 20–23, 28]. The follow up period varied from 5 months to 4.8 years. A substantial heterogeneity was observed in the applied cutoff values of TAPSE/PASP, which ranged from 0.27 to 0.58 mm/mmHg. With the exception of one study [19] all investigators consistently disclosed worst long-term event free survival for subjects with reduced values of TAPSE/PASP.

**Table 2:** Prognostic value of TAPSE/PASP ratio in heart failure (18 studies)

**Author**	**Year**	**Included in meta-analysis**	**HF population**	**Primary outcome**	**TAPSE/PASP cutoff value (mm/mmHg)**	**Follow up**	**Predictive value of TAPSE/PASP**
Guazzi et al. [5]	2013	Yes	HFrEF andHFpEF	Cardiovascular mortality	0.36	20 months (median)	TAPSE/PASP <0.36 was associated with worse event free survival
Guazzi et al. [28]	2015	No	HFrEF and HFpEF	Composite of cardiovascular mortality, left ventricular assist device implant, or heart transplant	0.40	4 years max	TAPSE/PASP <0.40 was associated with worse event free survival, Hazard ratio: 5.6 (3.5-8.9; p<0.001)
Iacoviello et al. [6]	2017	Yes	Stable HF, LVEF <45%	All-cause mortality	NA	36 months (range; 10-62)	After adjustment, TAPSE/PASP remained significantly associated with events in echocardiographic (HR: 0.69; 95% CI: 0.52–0.93; p: 0.016) but not in the clinical multivariate model (HR: 0.94; 95% CI: 0.71–1.25; p: 0.68)
Guazzi et al. [23]	2017	No	HFpEF	All-cause death and any cardiovascular hospitalization	0.35	13.4 months (range; 5.2-23.7)	Adverse outcomes were more common in the lower tertile (TAPSE/PASP <0.35) compared to the others
Ghio et al. [14]	2017	Yes	HFrEF and HFpEF	All-cause mortality	0.36	5 months (median)	Patients with TAPSE/PASP <0.36 and LVEF <40% demonstrated the worst long term event free survival
Bosch et al. [20]	2017	Yes	HFrEF and HFpEF	All-cause mortality and HF hospitalization	0.48	715 days (median)	Significant increase in the composite endpoint among patients with TAPSE/PASP <0.48 (log-rank p< 0.001)
Gorter et al. [18]	2018	Yes	HF with LVEF ≥45% and suspected pulmonary hypertension	All cause mortality	0.36	816 days (range; 547–1047)	TAPSE/PASP <0.36 was associated with worse event free survival (log-rank p=0.006)
Santas et al. [24]	2019	No	Acute HFpEF	Hospitalization for any cause	0.36	2 years (range; 0.74-3.6)	TAPSE/PASP <0.36 was associated with a higher risk of HF-related recurrent admissions (incidence rate ratio [IRR] 1.51, 95% CI, 1.01 to 2.24; p=0.040)
Falletta et al. [15]	2019	Yes	Clinically stable HFrEF	All-cause mortality	0.36	32 months (range; 20-53)	TAPSE/PASP ratio <0.36 had a threefold decrease in risk of death compared to the TAPSE/PASP ratio ≥0.36 group
Santas et al. [16]	2020	Yes	Acute HFpEF	All-cause mortality	0.36	1 year	The cohort with TAPSE/PASP <0.36 and significant tricuspid regurgitation had the highest cardiovascular mortality rates
Braganca et al. [19]	2020	Yes	HFrEF undergoing CRT	All-cause mortality	0.43	4 years (maximum)	No significant differences in survival were observed between groups with different RV-PA coupling (TAPSE/PASP <0.43 vs **≥**0.43: four-year survival of 80% vs 76%, p=0.72)
Rosa et al. [26]	2020	No	HFmrEF and HFrEF	All-cause mortality	0.34	25.5months (range; 8-46)	Survival free from all-cause mortality in patients with TAPSE/PASP <0.34 was worse as compared to that of patients with TAPSE/PASP ≥0.34 (log-rank p<0.001)
Palazzuoli et al. [7]	2020	No	Acute HFrEF and HFpEF	All-cause death and rehospitalization due to cardiovascular causes	0.43	6 months	TAPSE/PASP ratio<0.43 was related to increased risk of the outcome in univariable (HR: 2.31; CI 1.54–3.46; p<0.001) but not in the multivariable analysis
Schmeisser et al. [27]	2021	No	HFrEF with indication for CRT	All-cause mortality	0.38	4.8 years (median)	Patients with TAPSE/PASP <0.38 had significantly worse overall survival
Karam et al. [9]	2021	No	Functional MR undergoing TMVR with MitraClip	All-cause mortality	0.27	476 days (range; 225-727)	Survival rates at 1 and 2 years were lower among patients with impaired RV-PA coupling; 70.2 vs. 84.0%, respectively; p<0.001; and 53.4% vs. 73.1%, respectively; p<0.001)
Deaconu et al. [22]	2021	Yes	HFrEF undergoing CRT	All-cause mortality and HF rehospitalization	0.58	31 months (range; 18.1-43.9)	TAPSE/PASP<0.58 was associated with a higher risk of death or HF hospitalizations (HR 5.37; 95% CI 1.6-18; p<0.001)
Ishiwata et al. [21]	2021	Yes	Dilated cardiomyopathy, HFrEF	LV assist device implantation and all-cause death	-	12 months	After adjusting for age, BMI, NYHA class, systolic blood pressure and heart rate, TAPSE/PASP was independently associated (HR: 0.19; CI 0.03-0.82; p=0.02) with the outcome
Stassen et al. [8]	2022	Yes	HFrEFundergoint CRT	All-cause mortality	0.45	97 months (range; 54-143)	Survival rates at 5 years follow-up were significantly lower for patients with a TAPSE/PASP ratio <0.45 compared to those with a TAPSE/PASP ratio ≥0.45 (58 vs 82%, p<0.001)

*BMI* body mass index, *CI* confidence interval, *CRT* cardiac resynchronization therapy, *HF* heart failure, *HFmrEF* heart failure mildly reduced ejection fraction, *HFpEF* heart failure preserved ejection fraction, *HFrEF* heart failure reduced ejection fraction, *HR* hazard ratio, *LVEF* left ventricular ejection fraction, *NA* not available, *NYHA* new york heart association, *TAPSE/PASP* tricuspid annular plane systolic excursion/pulmonary artery systolic pressure, *TMVR* transcatheter mitral valve repair, *TR* tricuspid regurgitation, *TTVR* transcatheter tricuspid valve repair

### Quantitative analysis

#### Association of TAPSE/PASP ratio with all-cause death

**Fig. 2:**
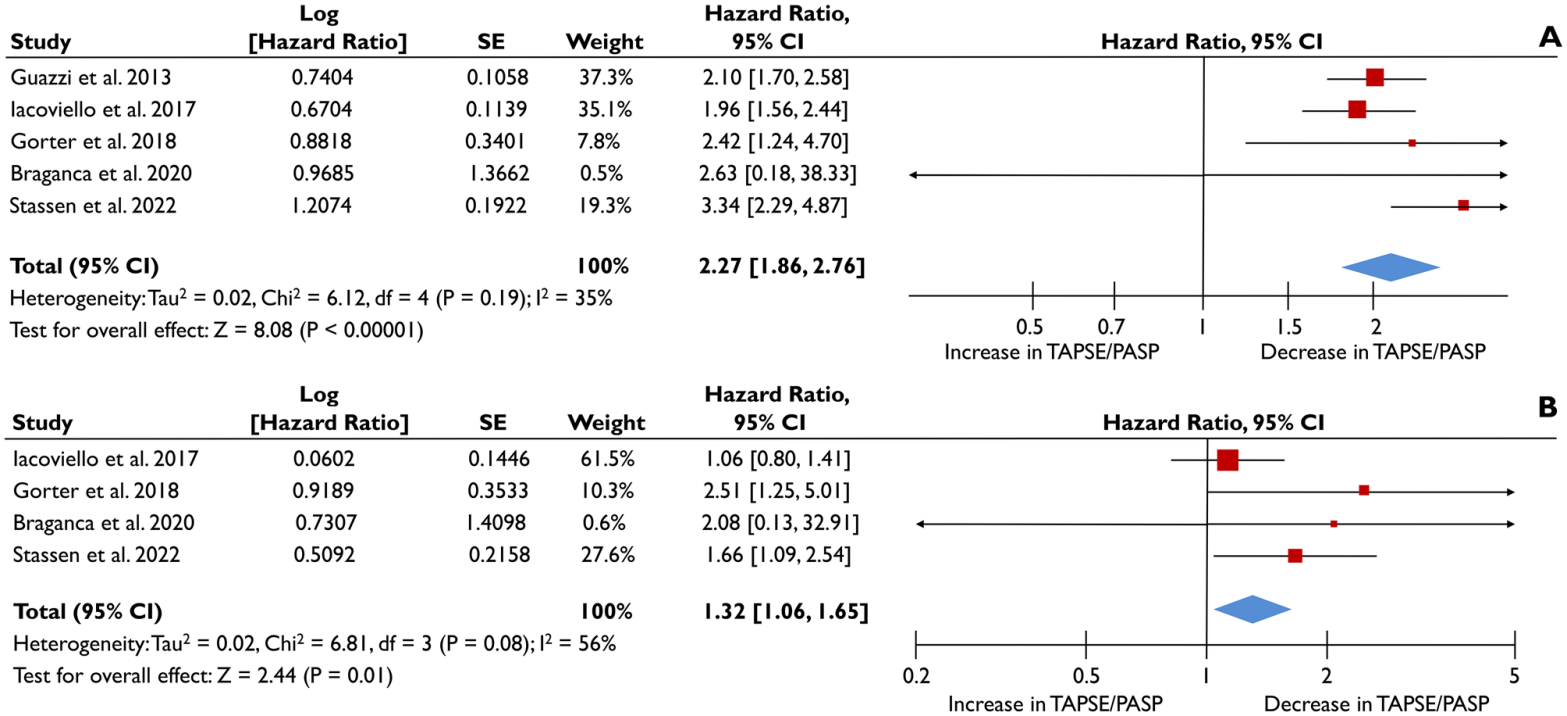
Association of TAPSE/PASP as continuous variable with all-cause death. The forest plots display the unadjusted (**A**) and adjusted (**B**) hazard ratios (HRs) and the corresponding 95% confidence interval, indicating the association of the tricuspid annular systolic plane excursion/pulmonary artery systolic pressure (TAPSE/PASP) ratio with all-cause death, as continuous variable in heart failure patients

A total of 5 studies provided appropriate data to quantitatively synthesize the association of TAPSE/PASP ratio, as a continuous variable, with all-cause death [5, 6, 8, 18, 19]. The pooled unadjusted HR was 2.27 (1.86-2.27; p<0.001;I^2^=35%) per 1 mm/mmHg reduction of TAPSE/PASP, as depicted in Fig. 2Α. When adjusted for pre-specified clinically-relevant parameters, it was shown that for each unit of reduction in TAPSE/PASP the risk for all-cause death was increased by 32% (pooled aHR=1.32 [1.06-1.65]; p=0.01; I^2^=56%) (Fig. 2Β).

**Fig. 3:**
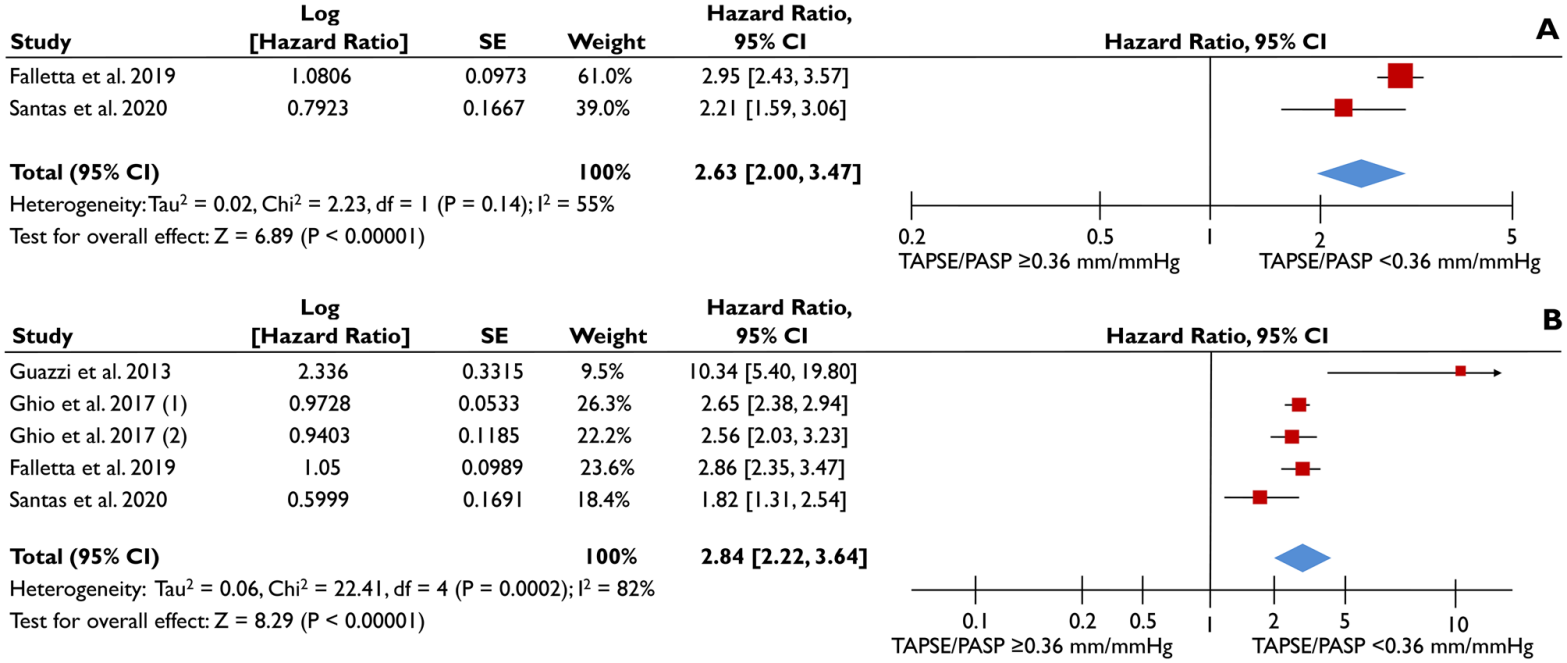
Association of TAPSE/PASP as categorical variable with all-cause death. The forest plots display the unadjusted (**A**) and adjusted (**B**) hazard ratios (HRs) and the corresponding 95% confidence interval, indicating the association of the tricuspid annular systolic plane excursion/pulmonary artery systolic pressure (TAPSE/PASP) ratio with all-cause death, as a categorical variable (cutoff value of 0.36 mm/mmHg applied) in heart failure patients. Ghio et al. provides different HRs for patients with left ventricular ejection fraction of <40% (1) or ≥40% (2) and, therefore, the 2 HRs have been separately included

Similar results were obtained when TAPSE/PASP ratio was assessed as a categorical variable, using the cutoff value of 0.36 mm/mmHg, retrieved from 4 studies which provided appropriate data [5, 14–16]. In the unadjusted analysis, TAPSE/PASP <0.36 mm/mmHg was associated with a more than 2-fold increased risk (pooled unadjusted HR: 2.63 [2.00-3.47]; p<0.001; I^2^=55%) of all-cause death compared to TAPSE/PASP ≥0.36 mm/mmHg (Fig. 3Α). After adjustment for clinically relevant cofounders, this strong association of TAPSE/PASP <0.36 mm/mmHg with increased all-cause death was retained (pooled aHR=2.84 [2.22-3.64]; p<0.001; I^2^=82%) (Fig. 3Β).

### Association of TAPSE/PASP ratio with the composite outcome

**Fig. 4:**
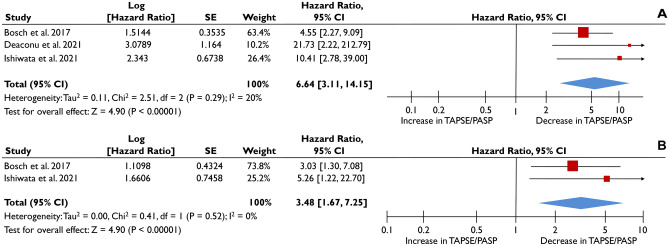
Association of TAPSE/PASP as continuous variable with the composite outcome. The forest plots display the unadjusted (**A**) and adjusted (**B**) hazard ratios (HRs) and the corresponding 95% confidence interval, indicating the association of the tricuspid annular systolic plane excursion/pulmonary artery systolic pressure (TAPSE/PASP) ratio with the composite outcome of all-cause death or heart failure hospitalization, as a continuous variable in heart failure patients

Regarding the composite outcome of all-cause death or HF hospitalization, a total of 3 studies provided obtainable data for meta-analysis [20–22]. The pooled unadjusted HR was 6.64 (3.11-14.15; p<0.001; I^2^=20%) per 1 mm/mmHg reduction in TAPSE/PASP, as illustrated in Fig. 4Α. After adjustment for cofounders, the results remained similar indicating that for each unit of reduction in TAPSE/PASP ratio the risk of the composite outcome was 3.48-fold higher (pooled aHR=3.48 [1.67-7.25]; p<0.001; I^2^=0%) (Fig. 4Β).

## Discussion

The present study was a systematic review and meta-analysis, encompassing a total 8,699 HF patients from a wide range of HF phenotypes, which examined the long-term prognostic value of non-invasive RV-PA coupling assessed with echocardiographic TAPE/PASP ratio. The quantitative synthesis confirmed the independent association of reduced TAPSE/PASP ratio, indicating worse RV-PA coupling, with increased all-cause death and worse composite outcome in HF cohorts. Additionally, the TAPSE/PASP ratio <0.36 mm/mmHg was independently associated with all-cause death in HF patients.

### Pathophysiology of RV-PA coupling

Left sided heart disease is by far the leading cause of pulmonary hypertension accounting for 65-80% of all cases [29]. Chronic elevation of the left-sided filling pressure due to systolic or diastolic dysfunction in HF is passively backwards transmitted. Elevated left atrial pressure is upstream transmitted to the pulmonary vasculature causing pulmonary vascular remodeling [30]. Pulmonary hypertension is ultimately transferred to the thin-walled flow-generator RV which is not designed to cope with brisk increases of pressure [31]. During the initial stages of pulmonary hypertension RV adapts by increasing its contractility to match the afterload excess and maintain pulmonary circulation [3]. However, as the disease progresses the compensatory phase ends leading to RV failure and disruption of the normal RV-PA coupling.

### Utility of TAPSE/PASP ratio

RV-PA uncoupling connotes an advanced stage of disease progression in left-sided heart disease, when trials to alter the course of disease with advanced therapies may be futile. In this regard, identifying such high-risk patients with ease in clinical practice is pivotal for risk stratification, but also to omit valueless procedures. Echocardiographic TAPSE/PASP ratio has emerged as an alternative predictor of invasive pressure-volume loop-derived end-systolic/arterial elastance, which is the gold standard measure of RV-PA coupling [32]. This index is the most extensively established surrogate of RV-PA coupling in HF [8, 23, 24], with fewer studies investigating alternative indices such as longitudinal strain of RV free wall/PASP [6, 33].

### RV-PA coupling for diverse HF cohorts

There is ample evidence that TAPSE/PASP ratio provides prognostic information beyond that provided from TAPSE or PASP separately in HF. Worse TAPSE/PASP values were associated with adverse long-term outcomes in all the 18 studies included in this systematic review, but 1 [19]. This strong association was demonstrated in various HF cohorts including outpatients with HF [15],patients in the acute phase of decompensation [24], subjects with HF with preserved [16] or reduced [27] ejection fraction, as well as recipients of CRT [8] or transcatheter valve procedures [9].

A substantial heterogeneity was observed in the definition of RV-PA uncoupling, with suggested TAPSE/PASP cutoff values varying from 0.27 to 0.58 mm/mmHg. Six studies [5, 14–16, 18, 24] implemented the cutoff of 0.36 mm/mmHg, initially proposed by Guazzi et al. [5] as derived by receiver-operating characteristic curve analysis from a mixed cohort of 293 HF patients. This cutoff value was strongly linked with all-cause death in the current meta-analysis (Fig. 3).

In one of the largest series of patients with acute HF and preserved ejection fraction, Santas et al. disclosed that TAPSE/PASP <0.36 mm/mmHg could independently predict unplanned rehospitalization for HF [24]. The risk was increased in a stepwise manner, with lower quintiles of TAPSE/PASP [24]. When the same cutoff value was applied for clinically stable HF patients with reduced ejection fraction, it demonstrated a threefold increase in the risk of death. On the contrary, Braganca et al. failed to elicit prognostic information for HF patients with reduced ejection fraction undergoing CRT, but their study was small and underpowered; a total of 70 patients were included with only 15 events [19]. Overall, TAPSE/PASP appears to be an efficient risk stratification tool for diverse HF phenotypes, including acute and chronic HF regardless of ejection fraction, as described by most studies and confirmed by the current quantitative analysis (Figs. 2, 3 and 4).

### RV-PA coupling as triage tool for interventional HF therapies

Contemporary evidence demonstrates than TAPSE/PASP can potentially identify non-responders to interventional HF therapies. Stassen et al. illustrated that among 807 CRT recipients TAPSE/PASP <0.45 mm/mmHg provided incremental prognostic information on top of impaired TAPSE [8]. In addition, lack of improvement in TAPSE/PASP ratio after CRT was associated with worst survival [8]. Those findings exemplify that when RV-PA uncoupling is established, the disease stage is so advanced that CRT implantation might come with little clinical benefit for the patient. A similar message was delivered by Karam et al., who elegantly demonstrated that patients with functional MR and pre-operative RV-PA uncoupling, defined as TAPSE/PASP <0.27 mmHg, derived significantly less survival benefit from transcatheter mitral valve repair compared to their counterparts [9].

Non-invasive TAPSE/PASP appears applicable for variable HF cohorts, however, in patients with greater than severe tricuspid regurgitation, it might overestimate the true coupling of the RV-PA circuit, as echocardiographic PASP is underestimated and correlates poorly with the respective invasive PASP. In a cohort of 126 patients with greater than severe tricuspid regurgitation undergoing transcatheter repair, only the TAPSE/PASP using the invasive PASP could provide prognostic information, whereas the echocardiographic TAPSE/PASP could not [10].

Overall, after excluding the patients with massive or torrential tricuspid regurgitation where echocardiographic PASP may not reflect the true pulmonary pressure, non-invasive TAPSE/PASP ratio can identify advanced HF phenotypes, where any potential benefit derived from intervention is attenuated.

### Future perspectives

The current systematic review and meta-analysis highlighted that the available observational data are more than enough to infer that RV-PA uncoupling, estimated from the reduced echocardiographic TAPSE/PASP ratio, connotes a poor prognostic course for HF patients receiving medical and interventional management. In order to favorably implement this index in clinical practice it is of paramount importance to consolidate a specific cutoff value; however different optimal cutoff values may be appropriate in different HF phenotypes (acute vs chronic, preserved vs reduced LV ejection fraction), and further research is warranted towards this direction. Ultimately, RV-PA uncoupling could serve as part of the standard HF risk assessment to select appropriate candidates that will benefit from advanced HF treatments including CRT or transcatheter valve repair of functional MR or tricuspid regurgitation. Thus, more studies have to be conducted in this direction of advanced interventional treatment of HF.

### Limitations

Although the current study follows a strict study selection protocol with a robust methodology some limitations should be acknowledged. First and foremost, the limitations of the present review are inherent to the observational nature of the included studies. The variations in the inclusion and exclusion criteria, endpoints reported, and cutoff values of TAPSE/PASP used contribute to the heterogeneity among studies. Subsequently, quantitative synthesis was limited to 11 out of the 20 studies included in the qualitative analysis.

With respect to the meta-analysis, a meta-regression analysis was unobtainable, and it was unfeasible to derive a universal cutoff value of TAPSE/PASP due to high discrepancy and limited reports of diagnostic accuracy data. Although the variables used for multivariate adjustment across the included in the meta-analysis studies coincide to some extent, they are not identical; hence pooled (a)HRs should be interpreted with caution.

## Conclusion

In summary, TAPSE/PASP ratio is a universally applicable non-invasive surrogate of RV-PA coupling that could be evaluated as part of the routine echocardiographic assessment of HF patients, to risk stratify them throughout the diverse HF phenotypes, since its reduction has been demonstrated to be independently associated with adverse outcomes. Future research should explore optimal cutoff values of the TAPSE/PASP ratio to define RV-PA uncoupling and ultimately integrate this index in HF risk stratification schemes for advanced interventional HF treatments.

### Supplementary Information

Below is the link to the electronic supplementary material.

Supplementary file1 (DOCX 24 kb)

## Data Availability

The data that support the findings of this study are available from the corresponding author, VK, upon reasonable request.

## Abbreviations

CIs: confidence intervals
CRT: cardiac resynchronization therapy
HF: heart failure
HR: hazard ratio
LV: left ventricle
MR: mitral regurgitation
PASP: pulmonary artery systolic pressure
RV: right ventricular-pulmonary artery
TAPSE: tricuspid annular systolic plane excursion
