# Supplementary material for: The prognostic impact of right ventricular-pulmonary arterial coupling in heart failure: a systematic review and meta-analysis
Source: Heart Fail Rev. 2023 Aug 28;29(1):13–26. doi: 10.1007/s10741-023-10341-2 (PMC10904417; doi:10.1007/s10741-023-10341-2)
Supplement: Supplementary file 1 — Supplementary file1 (DOCX 24 kb) [file 10741_2023_10341_MOESM1_ESM.docx]

**TITLE PAGE**

**Title:** The prognostic impact of right ventricular-pulmonary arterial coupling in heart failure: A systematic review and meta-analysis

**Running Title:** RV-PA coupling in heart failure

**SUPPLEMENTAL MATERIAL**

**Supplemental Table S1**. **Prisma checklist**

| **Section and Topic** | **Item #** | **Checklist item** | **Location where item is reported** |
| --- | --- | --- | --- |
| **TITLE** | | |  |
| Title | 1 | Identify the report as a systematic review. | Title, page 1 |
| **ABSTRACT** | | |  |
| Abstract | 2 | See the PRISMA 2020 for Abstracts checklist. | Abstract, page 2 |
| **INTRODUCTION** | | |  |
| Rationale | 3 | Describe the rationale for the review in the context of existing knowledge. | Page 4 |
| Objectives | 4 | Provide an explicit statement of the objective(s) or question(s) the review addresses. | Page 4 |
| **METHODS** | | |  |
| Eligibility criteria | 5 | Specify the inclusion and exclusion criteria for the review and how studies were grouped for the syntheses. | Page 5: “Study selection – Eligibility criteria” |
| Information sources | 6 | Specify all databases, registers, websites, organisations, reference lists and other sources searched or consulted to identify studies. Specify the date when each source was last searched or consulted. | Page 5: “Search strategy” |
| Search strategy | 7 | Present the full search strategies for all databases, registers and websites, including any filters and limits used. | Page 5: “Search strategy” |
| Selection process | 8 | Specify the methods used to decide whether a study met the inclusion criteria of the review, including how many reviewers screened each record and each report retrieved, whether they worked independently, and if applicable, details of automation tools used in the process. | Page 5: “Study selection – Eligibility criteria” |
| Data collection process | 9 | Specify the methods used to collect data from reports, including how many reviewers collected data from each report, whether they worked independently, any processes for obtaining or confirming data from study investigators, and if applicable, details of automation tools used in the process. | Page 6: “Data extraction” |
| Data items | 10a | List and define all outcomes for which data were sought. Specify whether all results that were compatible with each outcome domain in each study were sought (e.g. for all measures, time points, analyses), and if not, the methods used to decide which results to collect. | Page 6: “Outcomes of interest” |
|  | 10b | List and define all other variables for which data were sought (e.g. participant and intervention characteristics, funding sources). Describe any assumptions made about any missing or unclear information. | Page 6: “Data extraction” |
| Study risk of bias assessment | 11 | Specify the methods used to assess risk of bias in the included studies, including details of the tool(s) used, how many reviewers assessed each study and whether they worked independently, and if applicable, details of automation tools used in the process. | Page 7: “Risk of bias of individual studies” |
| Effect measures | 12 | Specify for each outcome the effect measure(s) (e.g. risk ratio, mean difference) used in the synthesis or presentation of results. | Page 7: “Data synthesis and statistical analysis” |
| Synthesis methods | 13a | Describe the processes used to decide which studies were eligible for each synthesis (e.g. tabulating the study intervention characteristics and comparing against the planned groups for each synthesis (item #5)). | Page 7: “Data synthesis and statistical analysis” |
|  | 13b | Describe any methods required to prepare the data for presentation or synthesis, such as handling of missing summary statistics, or data conversions. | Page 7: “Data synthesis and statistical analysis” |
|  | 13c | Describe any methods used to tabulate or visually display results of individual studies and syntheses. | Page 7: “Data synthesis and statistical analysis” |
|  | 13d | Describe any methods used to synthesize results and provide a rationale for the choice(s). If meta-analysis was performed, describe the model(s), method(s) to identify the presence and extent of statistical heterogeneity, and software package(s) used. | Page 7: “Data synthesis and statistical analysis” |
|  | 13e | Describe any methods used to explore possible causes of heterogeneity among study results (e.g. subgroup analysis, meta-regression). | Page 7: “Data synthesis and statistical analysis” |
|  | 13f | Describe any sensitivity analyses conducted to assess robustness of the synthesized results. | Page 7: “Data synthesis and statistical analysis” |
| Reporting bias assessment | 14 | Describe any methods used to assess risk of bias due to missing results in a synthesis (arising from reporting biases). | n/a |
| Certainty assessment | 15 | Describe any methods used to assess certainty (or confidence) in the body of evidence for an outcome. | n/a |
| **RESULTS** | | |  |
| Study selection | 16a | Describe the results of the search and selection process, from the number of records identified in the search to the number of studies included in the review, ideally using a flow diagram. | Pages7-8: “Search Outcomes” +Figure 1 |
|  | 16b | Cite studies that might appear to meet the inclusion criteria, but which were excluded, and explain why they were excluded. | Pages 7-8: “Search Outcomes” |
| Study characteristics | 17 | Cite each included study and present its characteristics. | Pages 7-8: “Search Outcomes” + Page 8: “Study characteristics”+ Table 1 |
| Risk of bias in studies | 18 | Present assessments of risk of bias for each included study. | Suppl Table S2 |
| Results of individual studies | 19 | For all outcomes, present, for each study: (a) summary statistics for each group (where appropriate) and (b) an effect estimate and its precision (e.g. confidence/credible interval), ideally using structured tables or plots. | Table 1 |
| Results of syntheses | 20a | For each synthesis, briefly summarise the characteristics and risk of bias among contributing studies. | Pages 9-10: “Quantitative analysis” + +Fig. 2-3 |
|  | 20b | Present results of all statistical syntheses conducted. If meta-analysis was done, present for each the summary estimate and its precision (e.g. confidence/credible interval) and measures of statistical heterogeneity. If comparing groups, describe the direction of the effect. | Pages 9-10: “Quantitative analysis” + +Fig. 2-3 |
|  | 20c | Present results of all investigations of possible causes of heterogeneity among study results. | Pages 9-10: “Quantitative analysis” + +Fig. 2-3 |
|  | 20d | Present results of all sensitivity analyses conducted to assess the robustness of the synthesized results. | n/a |
| Reporting biases | 21 | Present assessments of risk of bias due to missing results (arising from reporting biases) for each synthesis assessed. | n/a |
| Certainty of evidence | 22 | Present assessments of certainty (or confidence) in the body of evidence for each outcome assessed. | n/a |
| **DISCUSSION** | | |  |
| Discussion | 23a | Provide a general interpretation of the results in the context of other evidence. | Pages 10-13 |
|  | 23b | Discuss any limitations of the evidence included in the review. | Page 14: “Limitations” |
|  | 23c | Discuss any limitations of the review processes used. | Page 14: “Limitations” |
|  | 23d | Discuss implications of the results for practice, policy, and future research. | Pages 13-15: “Future Perspectives” + “Conclusion” |
| **OTHER INFORMATION** | | |  |
| Registration and protocol | 24a | Provide registration information for the review, including register name and registration number, or state that the review was not registered. | Page 5 |
|  | 24b | Indicate where the review protocol can be accessed, or state that a protocol was not prepared. | Page 5 |
|  | 24c | Describe and explain any amendments to information provided at registration or in the protocol. | n/a |
| Support | 25 | Describe sources of financial or non-financial support for the review, and the role of the funders or sponsors in the review. | n/a |
| Competing interests | 26 | Declare any competing interests of review authors. | n/a |
| Availability of data, code and other materials | 27 | Report which of the following are publicly available and where they can be found: template data collection forms; data extracted from included studies; data used for all analyses; analytic code; any other materials used in the review. | n/a |

**Supplemental Table S2: Studies of the systematic review not included in the meta-analysis**

| **Author** | **Year** | **Reason for exclusion** |
| --- | --- | --- |
| Guazzi et al. | 2015 | High risk of bias for study confounding and statistical analysis and reporting |
| Guazzi et al. | 2017 | Cox Proportional Hazards Analysis based on tertiles of TAPSE/PASP and no hazard ratio available for TAPSE/PASP as a continuous variable |
| Santas et al. | 2019 | Cox Proportional Hazards Analysis based on quartiles of TAPSE/PASP and no hazard ratio available for TAPSE/PASP as a continuous variable |
| Karam et al. | 2020 | Cox Proportional Hazards Analysis based on a cutoff value of TAPSE/PASP of 0.274 mm/mmHg and no hazard ratio available for TAPSE/PASP as a continuous variable |
| Rosa et al. | 2020 | High risk of bias for study confounding and statistical analysis and reporting |
| Schmeisser et al. | 2020 | High risk of bias for study confounding and statistical analysis and reporting |
| Palazzuoli et al. | 2020 | Cox Proportional Hazards Analysis based on a cutoff value of TAPSE/PASP of 0.425 mm/mmHg and no hazard ratio available for TAPSE/PASP as a continuous variable |

Abbreviations: TAPSE/PASP; tricuspid annular plane systolic excursion/pulmonary artery systolic pressure

**Supplemental Table S3: Risk of bias**

| Author | Year | Study Participation | Study Attrition | Prognostic Factor Measurement | Outcome Measurement | Study Confounding | Statistical analysis and reporting | Overall assessment |
| --- | --- | --- | --- | --- | --- | --- | --- | --- |
| Guazzi et al. | 2013 | L | L | L | L | M | L | L |
| Guazzi et al. | 2015 | L | M | M | L | H | H | H |
| Ghio et al. | 2017 | L | L | M | L | M | L | L |
| Iacoviello et al. | 2017 | M | M | L | L | M | M | M |
| Gorter et al. | 2017 | L | L | L | L | M | L | L |
| Bosch et al. | 2017 | L | L | M | L | L | L | L |
| Falletta et al. | 2019 | L | L | M | M | M | M | M |
| Braganca et al. | 2020 | M | M | M | L | M | M | M |
| Rosa et al. | 2020 | L | M | H | M | H | Η | H |
| Schmeisser et al | 2020 | L | M | M | M | H | H | H |
| Santas et al. | 2020 | L | M | M | L | L | M | M |
| Deaconu et al. | 2021 | L | L | M | L | H | M | M |
| Ishiwata et al. | 2021 | L | L | L | L | M | M | L |
| Stassen et al. | 2022 | M | L | M | L | L | L | L |

The Quality in Prognosis Studies (QUIPS) tool evaluates the individual overall risk of bias of the included studies based on 6 different domains. Those consist of study participation, study attrition, prognostic factor measurement, outcome measurement, study confounding, statistical analysis and reporting

Abbreviations: H, high; L, low; M, moderate
